# Quality and quantity of dromedary camel DNA sampled from whole-blood, saliva, and tail-hair

**DOI:** 10.1371/journal.pone.0211743

**Published:** 2019-01-31

**Authors:** Hasan Alhaddad, Tasneem Maraqa, Suha Alabdulghafour, Huda Alaskar, Randa Alaqeely, Faisal Almathen, Bader H. Alhajeri

**Affiliations:** 1 Department of Biological Sciences, Kuwait University, Safat, Kuwait; 2 Department of Veterinary Public Health and Animal Husbandry, College of Veterinary Medicine, King Faisal University, Al-Hasa, Saudi Arabia; 3 The Camel Research Center, King Faisal University, Al-Hasa, Saudi Arabia; University of Iceland, ICELAND

## Abstract

Camels are livestock with unique adaptations to hot-arid regions. To effectively study camel traits, a biobank of camel DNA specimens with associated biological information is needed. We examined whole-blood, saliva (buccal swabs), and tail-hair follicle samples to determine which is the best source for establishing a DNA biobank. We inspected five amounts of each of whole-blood, buccal swabs, and tail-hair follicles in nine camels, both qualitatively via gel electrophoresis and quantitatively using a NanoDrop spectrophotometer. We also tested the effects of long term-storage on the quality and quantity of DNA, and measured the rate of degradation, by analyzing three buccal swab samples and 30 tail-hair follicles over a period of nine months. Good quality DNA, in the form of visible large size DNA bands, was extracted from all three sources, for all five amounts. The five volumes of whole-blood samples (20–100μl) provided ~0.4–3.6 μg, the five quantities of buccal swabs (1–5) produced ~0.1–12 μg, while the five amounts of tail-hair follicles (10–50) resulted in ~0.7–25 μg. No differences in the rate of degradation of buccal swab and tail-hair follicle DNA were detected, but there was clearly greater deterioration in the quality of DNA extracted from buccal swabs when compared to tail-hair follicles. We recommend using tail-hair samples for camel DNA biobanking, because it resulted in both an adequate quality and quantity of DNA, along with its ease of collection, transportation, and storage. Compared to its success in studies of other domesticated animals, we anticipate that using ~50 tail-hair follicles will provide sufficient DNA for sequencing or SNP genotyping.

## Introduction

Dromedary camels (*Camelus dromedarius* Linnaeus, 1758) are multi-purpose livestock, domesticated for their adaptations to survive, reproduce, and produce (e.g. milk and meat) in hot-arid environments [1]. The two main hot-desert adaptations in dromedary camels are both extreme thermal tolerance and their high level of water conservation [2].

Camels tolerate heat through the insulation provided by their coats, along with exhibiting variable body temperature (34–40°C) [3, 4]. Adaptations to aridity in camels include tolerating water loss [3], having high drinking capacity, producing low amounts of urine, minimizing respiratory water loss [5], and maintaining the volume and function of red blood cells regardless of the water content in the blood stream [6, 7]. The aforementioned adaptations make camels the livestock of choice in the hot deserts of Asia and Africa [8]. Camels are utilized for the production of milk [9], meat [10], as well as hide and skin [11]. Camels are also used as pack animals, for transportation, for racing competitions [12, 13], and beauty contests [14, 15].

So far, there have been no thorough investigation of the molecular basis of camels’ traits, including desert adaptions, behavioral attributes, aesthetic traits, athletic traits, and traits of economic importance [16]. The dromedary camel genome has been sequenced recently [17, 18], which opens the door to study the camel genomically. The reference camel genome enabled the identification of genes associated with white-spotting coat color [19], solid coat colors [20], and “tameness” [21]. The aforementioned studies employed an approach that consists of sequencing candidate genes [22]—this approach is limited in utility and does not allow for exploring the wide-range of camel phenotypes. The candidate gene approach can be applied only when the biology of the phenotype is fully understood and a prior knowledge of candidate genes and their functions is available [22]. A biobank of camel DNA samples, with associated phenotypes, is needed to utilize genome sequencing and SNP genotyping technologies in linkage analyses [23] or genome-wide association studies [24]. Moreover, validating the association and possibly the causation, of the genetic variants of a given phenotype requires genotyping a large number of individuals (positive and negative for the phenotype) and examining the agreement between their genotype and phenotype [25]. This validation method will benefit greatly from a camel DNA biobank.

DNA biobanking is essential to study livestock genetics and to develop controlled breeding programs [26, 27]. DNA biobanking for genetic studies requires careful consideration of the used biological specimens, insofar as their source, and their yielded quality and quantity to enable DNA genotyping and sequencing, as well as their ease of transport, and their amenability for long-term storage prior to DNA extraction. Another important aspect to consider when establishing a DNA biobank, is the depth of the information associated with the samples collected from each specimen. Such information includes age, sex, breed, pedigree information, geographic location, phenotypic attributes (e.g. coat color and texture), morphological measurements (e.g. size and height), and productivity (e.g. milk volume, carcass weight, hide quality). To this effect, we recently developed the data collection and organization application, *SamplEase* [28], that aims to expedite the collection of all the necessary information associated with each DNA sample in the envisioned camel biobank.

DNA sequencing for molecular studies were successful in using each of camel blood [17–20], tissue [29], saliva [30], and hair [19, 21]. However, the question of which DNA source is most useful for camel sample biobanking still remains. We think that for the DNA source to be optimal in camel biobanking, it should have the following attributes: (1) it should be easy and non-invasive to collect, (2) the collection should require no special training, (3) it should neither be pathogenic nor harmful, (4) it should provide adequate DNA quantity and quality, (5) it should be amenable to long-term storage, and (6) it should be easy to transport to the lab and distribute to interested researchers.

To select the best camel specimen source for establishing a DNA biobank, we aimed to (1) identify the most optimal, non-invasive method of camel DNA sampling, (2) assess the quantity and quality of DNA obtained from whole-blood, saliva (buccal swabs), and tail-hair follicles, and (3) examine the degradation rate of the DNA extracted from tail-hair and saliva, over a period of 9 months. The third aim was confined to saliva and tail-hair samples due to their nature of room-temperature storage prior to DNA extraction (unlike blood, which needs refrigeration).

## Materials and methods

### Ethics statement

Samples were collected in accordance with the “Guidelines for Ethical Conduct for Use and Care of Animals in Research” and approved by the Ethics Care Committee, King Faisal University (KFU), Saudi Arabia.

### Animals

Nine unrelated research camels housed at the Camel Research Center in King Faisal University in Saudi Arabia were used for the study. The camels represent three widely recognized camel breeds in the Arabian Peninsula (‘Majaheem’, ‘Sofor’, ‘Waddah’) [31].

### Sampling biological specimens

Samples were collected from healthy camels during routine veterinary medical examination. Whole-blood samples were obtained by restraining the camels in a laying down position (with the assistance of two camel handlers) and drawing 5 milliliters of whole-blood from the jugular vein using a sterile needle. The whole-blood samples were immediately placed into EDTA tubes, which were transferred to an ice-box for transport, and eventually stored at 4°C until the process of DNA extraction.

One hundred buccal swabs were used to collect saliva samples from each camel. Buccal swab samples were collected five at a time, by scrubbing the inner cheeks of each camel. To minimize the amount of camel feed in the saliva samples, camels’ mouths were washed with clean bottled water prior to sample collection. Swabs were then air-dried using a Styrofoam holder, prior to being placed in their sterile protective envelopes.

Hair follicle samples were collected from the tail by plucking them from their roots. Tail-hair tips were bundled (50 tips at a time), plucked, attached to a duct tape ~15 mm away from the follicles, and the hair tips were trimmed and discarded. The tail-hair bundles (retained in the duct tape) were then placed in a labeled envelope for storage. This process was repeated multiple times, with ~50 hairs being collected per bundle, resulting in ~500–1000 hairs per camel.

### DNA extraction

**Fig 1:**
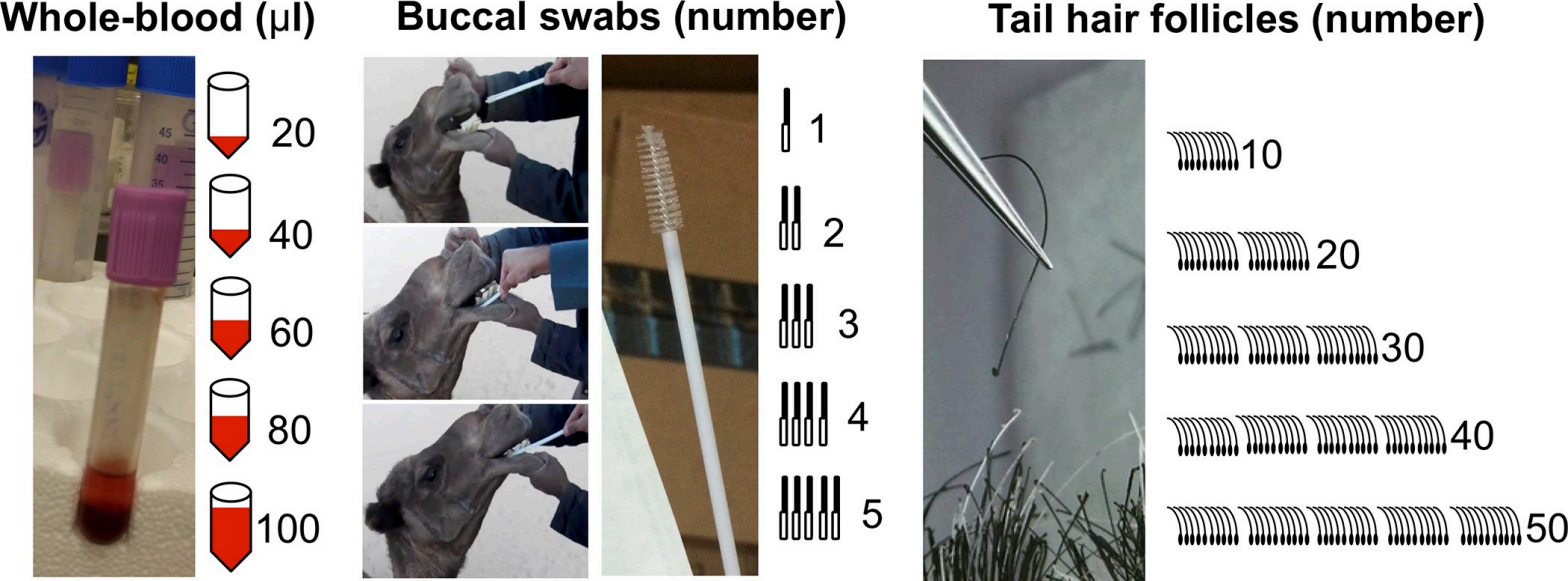
The three sources, and the five quantities, of the camel DNA samples used in the study. DNA extractions were performed on the five different quantities, of each one of the three DNA sources.

For each of the nine sampled camels, DNA was extracted from five quantities for each of the three sources: 20, 40, 60, 80, and 100μl of whole-blood; 1, 2, 3, 4, and 5 buccal swabs; and 10, 20, 30, 40, and 50 tail-hair follicles (Fig 1). Tail-hair follicles were manually separated and counted using a magnifying lens and a light source. To account for possible inconsistencies in DNA extractions due to natural differences in starting material, three separate extractions (i.e. replicas) were performed for each of the nine camels, for each of the three sources and the five abovementioned quantities.

DNA was extracted using a DNA extraction kit (PureLink Genomic DNA Mini Kit, Thermo Fisher Scientific), following the recommendations and protocol of the kit’s manufacturer. DNA was eluted using 100 μl of the elution buffer twice. Each of the two 100 μl elutions was placed in a separate tube. The purpose of having a second elution was to provide a diluted sample that can be used directly for a polymerase chain reaction (PCR) (No PCR was performed in the current study).

### DNA quality and quantity assessment

The quality of the DNA extracted from each of the samples was examined using gel electrophoresis. We mixed 5 μl from the first elution of each extracted DNA sample with an equal amount of loading buffer, before placing the mixture in a 1.5% agarose gel. The quality of the extracted DNA was assessed by a direct comparison of visibility and size of the extracted DNA to that of a lambda-HindIII or a 100 bp ladder. The quantity of each extracted sample (for both the first and the second elutions) was measured using spectrophotometry (single channel Nanodrop 1000), at the Biotechnology Center in Kuwait University.

### Statistical analysis of camel DNA quantities

Summary statistics of DNA quantity and purity parameters from each DNA source were calculated in R [32] and presented using the BEESWARM package [33]. A linear regression analysis was used to predict extracted DNA amount (of the first elution) based on starting sample quantity (μl of whole-blood, number of buccal swabs, number hair follicles)—separate regression analyses were conducted for each of the three DNA sample sources.

For each DNA source (whole-blood, saliva, and tail-hair), an analysis of covariance (ANCOVA) was conducted, with extracted DNA amount (of the first elution) modeled as the response variable and starting DNA sample quantity as the covariate, and each of ‘replica’, ‘individual camel’, and ‘breed’ as fixed factors (in separate ANCOVAs). These ANCOVAs allow us to compare the regression slopes divided by the fixed factors, to see if they are homogenous (thus testing for consistency of the relationship between starting sample quantity vs. extracted DNA amount of the first elution, across these fixed factors). Because some of the assumptions of the parametric linear regression and the parametric ANCOVA models were not met (i.e. normality and/or homoscedasticity), nonparametric (permutation-based) alternatives of both analyses were used, with statistical significance for each determined using a maximum of 100,000 iterations, as implemented in the LMPERM package [34].

The C1 camel was omitted for the whole-blood analysis because it was extracted once with no ‘replicas’ and only for 20, 40, 60, 80 μl (and not 100 μl) due to sample overuse in troubleshooting experiments. In addition, the C8 and the C9 camels were omitted from the analysis, because we discovered that incorrect reagents were used in the extraction protocol for these two camels following the experiments.

### DNA degradation analysis

For each of the nine sampled camels, DNA was extracted from three buccal swabs and 30 tail-hair follicles every three months, over a period of nine months. To account for inconsistencies between extractions, three replicas were performed for each of the camels and for each DNA source. The quality and quantity of the DNA obtained at each three-month period was examined using the methodology described above.

The rate of DNA degradation of saliva and tail-hair was assessed with a nonparametric linear regression analysis, where time of sample retrieval (i.e. each three-month period) was used to predict extracted DNA amount (of the first elution), with separate regression analyses conducted for each DNA sample source. In addition, for each sample of saliva and tail-hair, we conducted a separate nonparametric ANCOVA, with extracted DNA amount (of the first elution) modeled as the response variable and time as the covariate, and each of ‘replica’, ‘individual camel’, and ‘breed’ as fixed factors to compare the regression slopes divided by the fixed factors to see if the rate of DNA degradation is consistent across these fixed factors. The rate of DNA degradation of the saliva samples was compared to the tail-hair samples by comparing their overall regression slopes using a nonparametric ANCOVA. As above, the significance of all nonparametric linear regressions and ANCOVAs was based on a maximum of 100,000 iterations.

## Results

### DNA quality and quantity

The DNA extracted from all five-whole-blood quantities (20, 40, 60, 80,100 μl) was clearly visible on the gel, as a single, large-sized band, that corresponds to genomic DNA (S1 Fig). We detected no qualitative signs of DNA degradation (i.e. no smears) in all five quantities of the extracted whole-blood (S1 Fig). There were also no differences in the quality of DNA between the three replicated extractions of each individual camel, no quality differences across individuals within a breed, or across breeds, and no differences in the clarity (i.e. intensity) and position of the DNA bands from the five quantities of whole-blood (20–100 μl) (S1 Fig).

**Fig 2:**
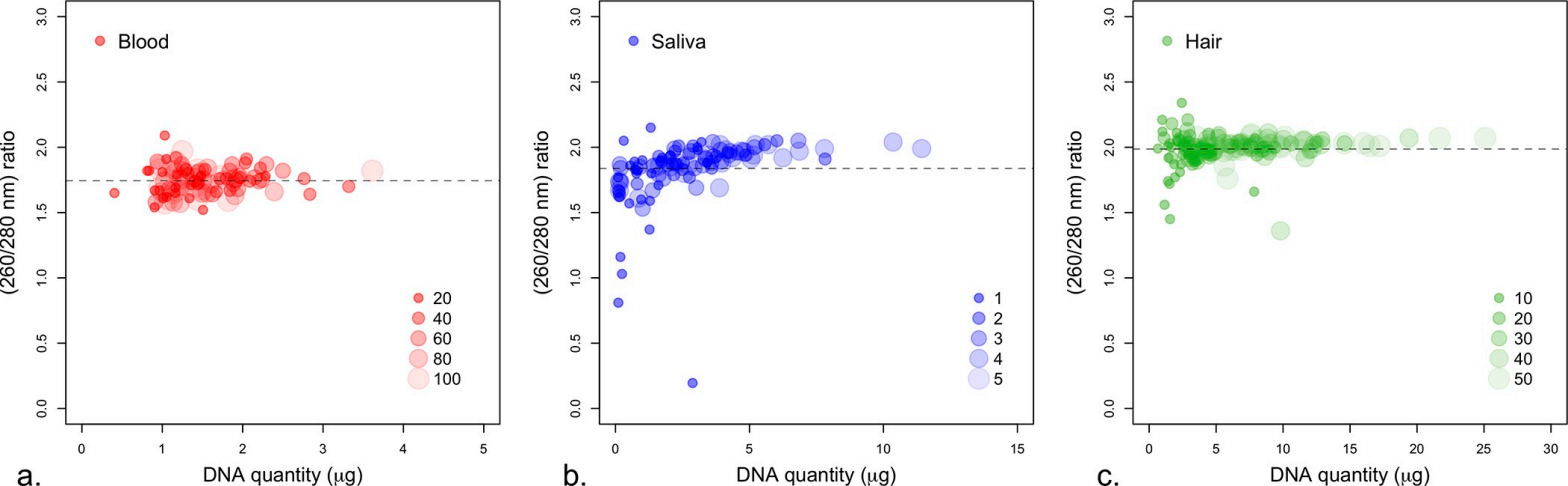
Quantity and purity of camel DNA extracted from whole-blood, saliva, and tail-hair follicles. (a-c) DNA quantity (μg) on the x-axis and 260/280 nm ratio on the y-axis from whole-blood, buccal swabs, and tail-hair follicles, respectively. Each circle corresponds to a measurement (DNA quantity and 260/280 nm ratio) from the first elution (E1 = 100μl) and the size of the circle represents the different amounts of starting material (blood = 20, 40, 60, 80, 100μl, saliva = 1, 2, 3, 4, 5 swabs, hair = 10, 20, 30, 40, 50 follicles). Dashed horizontal lines are the mean 260/280 nm ratio.

Quantitatively, the five whole-blood quantities (20, 40, 60,80, 100 μl) resulted in an overall range of 0.4–3.6 μg genomic DNA and a 1.52–2.53 range of 260/280 ratios (Fig 2A). The DNA amount from each individual whole-blood quantity (20, 40, 60,80, 100 μl) averaged to 1.05, 1.69, 1.53, 1.30, and 1.28 μg, respectively (Table 1; S2 Fig). On average the amount of DNA obtained from the second elution is approximately half (49.7%) of what was obtained from the first (Table 1; S2 Fig).

**Table 1:** Summary of DNA amounts (μg) obtained from five quantities of each of: whole-blood, buccal swabs (saliva), and tail-hair follicles.

Source	Starting material	N	Elution	Min.	Mean	Max.	S.D.
Blood	20	19	E1	0.40	1.13	2.28	0.38
		19	E2	0.30	0.50	0.95	0.15
	40	19	E1	1.17	1.89	3.32	0.57
		19	E2	0.50	0.78	1.13	0.18
	60	19	E1	0.92	1.71	2.05	0.48
		19	E2	0.42	0.83	1.14	0.21
	80	19	E1	0.99	1.45	2.40	0.36
		19	E2	0.42	0.74	1.13	0.17
	100	18	E1	0.96	1.44	3.61	0.62
		18	E2	0.40	0.74	1.16	0.18
Saliva	1	27	E1	0.10	1.30	3.42	1.04
		27	E2	0.05	0.45	1.80	0.35
	2	27	E1	0.11	2.67	7.82	1.82
		27	E2	0.08	0.74	2.14	0.45
	3	27	E1	0.16	2.89	6.83	1.67
		27	E2	0.11	0.92	2.07	0.52
	4	27	E1	0.14	4.1	11.43	2.83
		27	E2	0.10	1.22	2.87	0.74
	5	27	E1	0.13	5.21	12.04	3.58
		27	E2	0.09	1.41	3.12	0.79
Hair	10	27	E1	0.66	2.46	7.90	1.73
		27	E2	0.81	0.84	1.71	0.37
	20	27	E1	1.12	4.15	12.45	2.58
		27	E2	0.23	1.30	2.41	0.57
	30	27	E1	2.58	6.26	14.60	3.30
		27	E2	0.30	2.40	7.77	1.76
	40	27	E1	2.73	8.01	19.40	3.65
		27	E2	0.75	2.80	9.77	1.92
	50	27	E1	3.34	10.19	25.09	5.30
		27	E2	0.71	3.31	8.99	1.95

Notes: Amount = starting material (μl) whole-blood, number of buccal swabs, and number of tail-hair follicles. N = number of samples. Elution = the first (E1) or second (E2) elution from the DNA extraction column. Min, Mean, Max, and S.D. = minimum, mean, maximum and standard deviation of DNA amounts (μg), respectively.

When all the data were considered together, the nonparametric linear regression analysis indicated that starting whole-blood DNA sample quantity (20–100 μl) did not significantly predict the extracted DNA amount of the first elution (*p* = 0.403). The nonparametric ANCOVAs detected a significant interaction effect between starting whole-blood DNA sample quantity and the breed factor (*p* = 0.0285) but starting whole-blood DNA sample quantity did not interact with the replica factor (*p* = 0.2504) nor individual camel factor (*p* = 0.1317; S1 Table). These results indicate that the relationship between starting whole-blood DNA sample quantity and extracted DNA amount (of the first elution) is different among different camel breeds (heterogenous regression slopes) but is not different across both experimental replicates nor individual camels (homogenous regression slopes).

DNA extracted from the five buccal swab quantities (1, 2, 3, 4, and 5 swabs) was clearly visible on the gel as a single, large-sized band (S3 Fig). However, minor signs of DNA degradation were detected in the form of smears, in the extractions that used 3, 4, and 5 swabs (S3C–S3E Fig). Visible differences in DNA quality (i.e. band intensity, presence of a smear) were detected between the three replicated extractions of each individual, and between individuals but not among the breeds (S3 Fig). Also, visible differences in the intensity of the bands were observed for the various swab numbers, where the highest intensity bands were found for the largest amount of swabs (5 swabs) (S3E Fig).

Wide ranges of DNA amounts (0.1–12.04 μg) and 260/280 ratios (0.2–2.15) were obtained from the various swab quantities (Fig 2). DNA extracted from a single buccal swab had the largest variability in the 260/280 ratios, which may indicate a low purity. The saliva quantities (1,2 3, 4, and 5 buccal swab) averaged to 1.30, 2.67, 2.89, 4.10, and 5.21 μg, respectively—the second elution on average contained around 42.3% of the amount of DNA in the first elution (Table 1; S2 Fig).

When all the data were considered together, the nonparametric linear regression analysis indicated that starting saliva DNA sample quantity (1,2 3, 4, and 5 buccal swab) significantly predicted extracted DNA amount (of the first elution) (*p* < 0.0001, *R*^2^_adj_ = 0.23), indicating that 23% of the variation in the amount of extracted DNA (of the first elution) was accounted for by the variation in the starting DNA sample quantity. The nonparametric ANCOVAs detected a significant interaction between starting saliva DNA sample quantity and the individual camel factor (*p* < 0.0001) but starting saliva DNA sample quantity did not interact with the replica factor (*p* = 1.0000) nor with the breed factor (*p* = 0.0912; S2 Table). These results indicate that the relationship between starting saliva DNA sample quantity and extracted DNA amount (of the first elution) is different among different individual camels but is not different across both experimental replicates nor camel breeds.

Qualitatively, DNA extracted from the five tail-hair follicle quantities (10, 20, 30, 40, 50 hair follicles) showed different patterns with regard to starting material and resulting DNA band intensity on agarose gels to that of whole-blood and saliva (S4 Fig). DNA extracted from 10 tail-hair follicles was barely visible (S4A Fig), whereas DNA extracted from each of: 20, 30,40, 50 tail-hair follicles was clearly visible on the gel, as a single, large-sized band for each treatment (S4B–S4E Fig). Minor signs of DNA degradation were detected in the form smears, especially for the extractions that used 40 and 50 follicles (S4D and S4E Fig). Increased tail-hair follicle quantities were associated higher extracted DNA band intensities (S4 Fig), Implying increased DNA amount. No differences in the quality of DNA were detected between the three replicated extractions of each individual, among individual camels, or among breeds (S4 Fig).

The different numbers of tail-hair follicles resulted in a 0.66–25.08 μg range in extracted DNA amount and a range of 260/280 ratios of 1.36–2.34 (Fig 2C). The 10, 20, 30, 40, 50 hair follicles provided, on average, 2.46, 4.15, 6.26, 8.01, and 10.19 μg of extracted DNA, respectively, with an average 260/280 ratio of 1.99 (Table 1). The second elution on average contained ~37.7% of the amount of DNA in the first elution (Table 1; S2 Fig).

When all the data were considered together, the nonparametric linear regression analysis indicates that starting tail-hair DNA sample quantity (10, 20, 30, 40, 50 hair follicles) significantly predicted extracted DNA amount (of the first elution) (*p* < 0.0001, *R*^2^_adj_ = 0.38), indicating that 38% of the variation in the amount of extracted DNA of the first elution was accounted for by the variation in the starting DNA sample quantity. The nonparametric ANCOVAs detected a significant interaction effect between starting tail-hair DNA sample quantity and the individual camel factor (*p* = 0.0227) but starting tail-hair DNA sample quantity did not interact with the replica (*p* = 0.7296) nor with the breed factor (*p* = 0.7297; S3 Table). These results indicate that the relationship between starting tail-hair DNA sample quantity and extracted DNA amount (of the first elution) is different among different individual camels but is similar across both experimental replicates and camel breeds.

### Camel DNA degradation:

**Fig 3:**
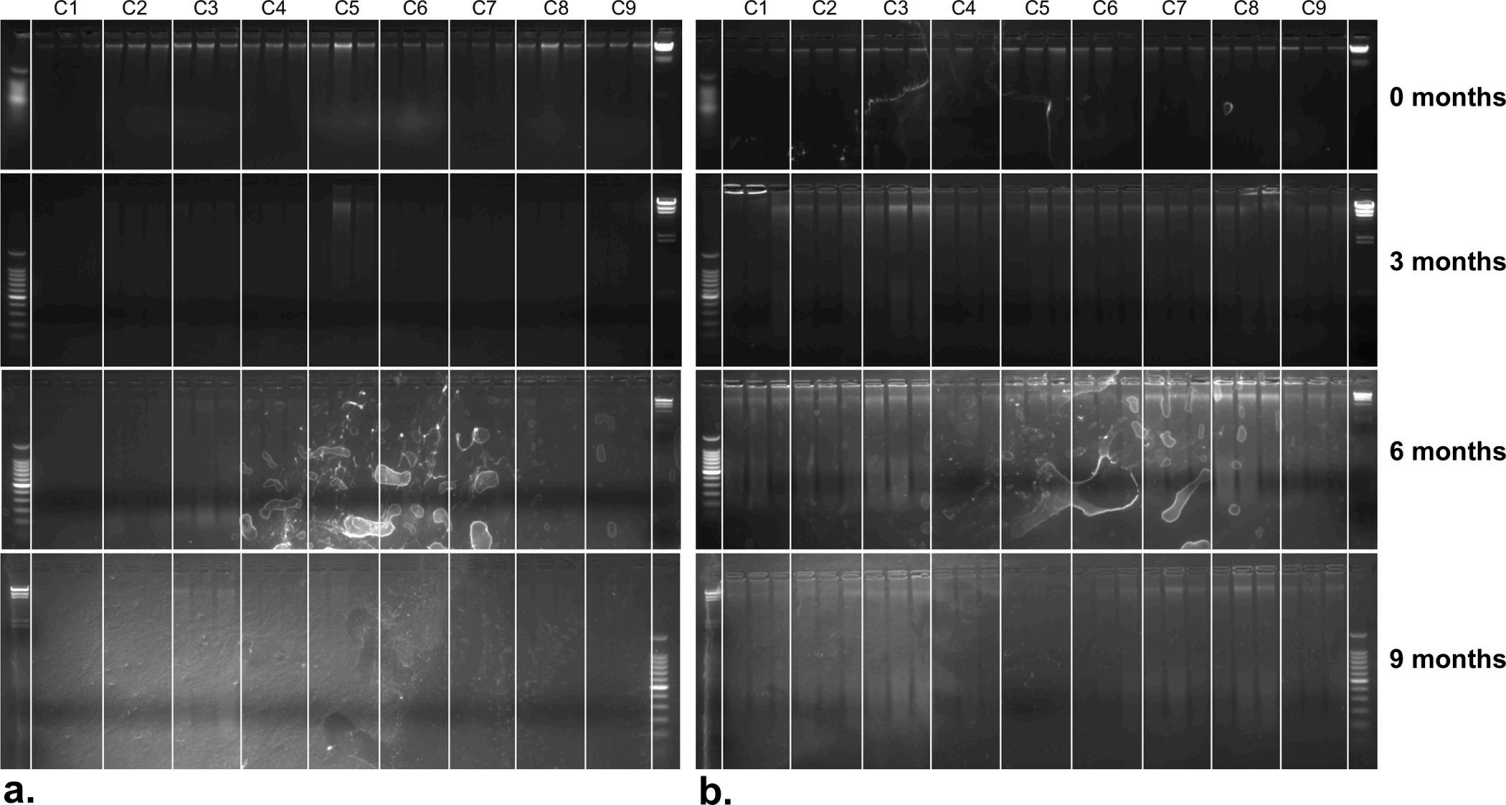
Electrophoretic analysis of the degradation of DNA extracted from buccal swabs and tail-hair follicles over a 9-month period. (a) 1.5% agarose gels of DNA extracted from three camel buccal swabs every three months, for a period of 9 months. (b) 1.5% agarose gels of DNA extracted from thirty camel tail-hair follicles every three months, for a period of 9 months. C1-C3: Majaheem, C4-C6: Sofor, C7-9: Waddah. Each buccal swab and tail-hair follicle quantity of each of the nine camels (C1-9) was extracted three times (replicas). The DNA in the gels is that of the first elution (E1). The two ladders used in the gels are 100 bp (left side) and lambda-HindIII molecular markers (right side). The ladders in the bottom gels are in reversed positions. Note: the six months gels contain marks but the presence and absence of large size DNA bands and smears are visible for comparison.

Extracted DNA from the buccal swabs was visible as large-sized band in the initial extraction (Fig 3A– 0 months). In all three subsequent extractions, over a 9-month period, the large-sized band (i.e. genomic DNA) was absent, and large smears were found on the gel, which resembled signs of degraded DNA (Fig 3A– 3, 6, and 9 months). In contrast, DNA extracted from tail-hair follicles, over all four extraction periods, showed a clear, large-sized band (Fig 3B). With the exception of the initial extraction, noticeable signs of degraded DNA (i.e. smears) were observed, with the observed smears being accompanied by clearly visible large-sized bands (Fig 3B).

**Fig 4:**
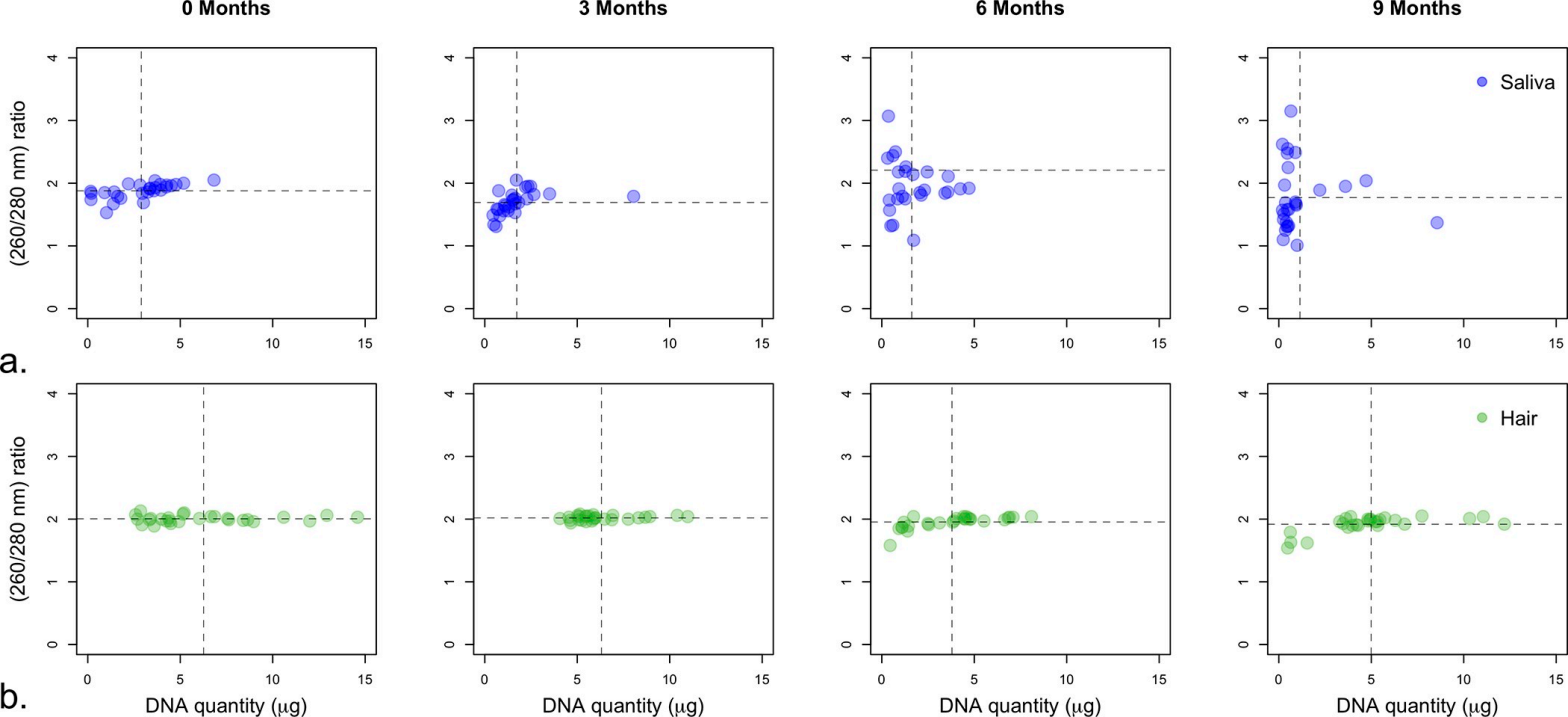
Quantity and purity of camel DNA extracted from saliva (buccal swabs) and tail-hair follicles over a nine-month period. DNA quantity (μg) on the x-axis and 260/280nm ratio on the y-axis over a nine-month period (0, 3, 6, and 9 month) extraction times, from (a) buccal swabs and (b) tail-hair follicles. Each circle corresponds to a single measurement (DNA quantity and 260/280nm ratio) from the first elution (100μl). The dashed horizontal line is the mean 260/280nm ratio and the dashed vertical line is the mean of the DNA quantities (μg).

Quantitatively, the buccal swab extractions for the four extraction times (0, 3, 6, and 9 months) resulted in an average of 2.89, 1.73, 1.62, and 1.14 μg of extracted DNA, respectively (Table 2, Fig 4A). At the same time, the tail-hair follicle extractions produced an average amount of DNA of 6.26, 6.31, 3.80, and 4.99 μg for the four extraction times (0, 3, 6, and 9 months) (Table 2, Fig 4B).

**Table 2:** Summary of DNA extracted from camel saliva and tail-hair samples over a nine-month period.

Source	Month	N	Elution	Min.	Mean	Max.	S.D.
Saliva	0	27	E1	0.16	2.89	6.83	1.67
		27	E2	0.11	0.92	2.07	0.52
	3	27	E1	0.44	1.73	8.05	1.46
		27	E2	0.13	0.59	1.15	0.26
	6	27	E1	0.31	1.62	4.72	1.29
		27	E2	0.12	0.49	1.54	0.35
	9	27	E1	0.20	1.14	8.56	1.81
		27	E2	0.02	0.27	0.93	0.25
Hair	0	27	E1	2.60	6.26	14.60	3.30
		27	E2	0.30	2.39	7.77	1.76
	3	27	E1	4.06	6.31	10.98	1.78
		27	E2	0.82	1.73	3.55	0.57
	6	27	E1	0.45	3.80	8.09	2.19
		27	E2	0.33	1.39	2.47	0.58
	9	27	E1	0.47	4.99	12.20	2.86
		27	E2	0.06	1.18	3.68	0.85

Notes: N = number of samples. Elution = the first (E1) or second (E2) elution from the DNA extraction column. Min, Mean, Max, and S.D. = minimum, mean, maximum and standard deviation of DNA amounts (μg), respectively.

Visually, there seems to be considerable differences in the 260/280 nm ratio (purity) in saliva samples especially for 6- and 9-month period extractions (Fig 4A, notice distribution over y-axis) whereas tail-hair samples showed similar ratios (Fig 4B).

When all the data were considered together, the nonparametric linear regression analysis indicates that time significantly predicted extracted DNA amount (of the first elution) for each of saliva (*p* = 0.0002, *R*^2^_adj_ = 0.12) and tail-hair (*p* = 0.0055, *R*^2^_adj_ = 0.06). This result indicates that time accounts for 12% of the variation in the amount of extracted DNA (of the first elution) for saliva and 6% of the variation in the amount of extracted DNA (of the first elution) for tail-hair.

For saliva samples, the nonparametric ANCOVAs detected a significant interaction effect between time and the individual camel factor (*p* = 0.0012) but time did not interact with the replica factor (*p* = 0.7789) nor with breed membership (*p* = 0.4008; S4 Table). These results indicate that the relationship between time and extracted DNA amount (of the first elution) is different among different individual camels, but is not different across both experimental replicates nor camel breeds.

For tail-hair samples, the nonparametric ANCOVAs detected a significant interaction effect between time and the individual camel factor (*p* < 0.0001) and the breed factor (*p* = 0.0163), but time did not interact with the replica factor (p = 0.6222; S5 Table). These results indicate that the relationship between time and extracted DNA amount (of the first elution) is different among different individual camels and among camel breeds but is similar experimental replicates.

**Fig 5:**
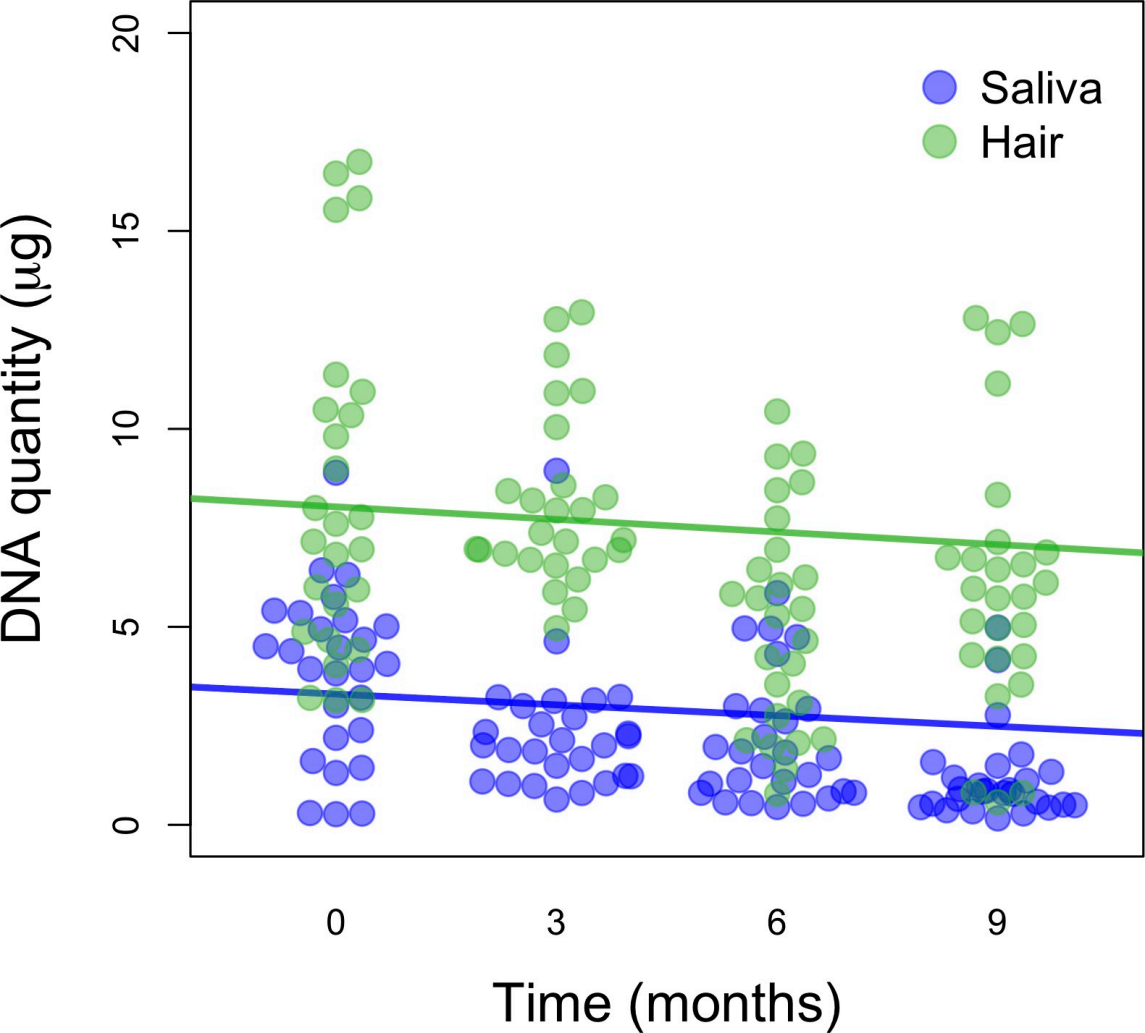
Scatterplot of time vs. extracted DNA amount (μg). Samples are separated by DNA source (saliva vs. tail-hair). The amount of DNA is that obtained from the first and second elutions combined (total). Best-fit lines are also shown for each DNA source.

The nonparametric ANCOVA detected no significant interaction effect between time and DNA source (*p* = 1.0000; S6 Table), indicating that the relationship between time and DNA source is not different overall (all data), or that the rate of DNA degradation was similar across saliva and tail-hair DNA sources. A visual inspection of a scatterplot of time vs. extracted DNA amount (of the first elution), separated by DNA source, shows that over all four three-month time periods, the amount of DNA extracted from hair was consistently greater than that from saliva (Fig 5).

## Discussion

In this study, we focused on camel DNA sources most suitable for establishing a DNA biobank. Whole-blood, saliva (buccal swabs), and tail-hair follicles were examined for ease of collection, storage, and transportation. A comparison of the quality and quantity of DNA extracted from each of these three sources was compared, with specific emphasis on their potential utility for subsequent genetic analyses (e.g. quantity, degradation tolerance).

### Collection, transportation and storage

Blood samples are the DNA source of choice for genetic studies, however, many disadvantages limit their usability for large-scale camel DNA biobanking [35–37]. These limitations are that camel blood collection requires (1) special veterinary training, (2) assistance of camel handlers, (3) collection tools (needles, syringes, EDTA tubes etc.), (4) care to avoid accidents due to contact (i.e. kicks, bites), (5) precaution against possible harmful pathogens [38, 39], (6) long camel-handling time, (7) breeder cooperation, and (8) special means of transportation and storage (i.e. coolers, freezers) [40]. By comparison, buccal swab collection only requires handling assistants, care to avoid accidental bites, sterile cytological brushes, and moderate collection time (personal observation). Tail-hair samples have the least requirements, where no special training or assistants are needed, no collection tools, and minimal camel handling time (leading to fast sample collection), and easy transportation and storage of specimens in envelopes at room temperature (personal observation). These minimal requirements for tail-hair collection makes them more suitable than whole-blood and buccal swabs for establishing a DNA biobank, as recognized for other domesticated animals such as cattle and yaks [36].

### DNA quality and quantity

Overall high genomic DNA quality in the form of a visible, large-sized, and intact, single band was obtained from all three sources in camels, even with as little as 20μl of whole-blood, a single buccal swab, or 10 tail-hair follicles (S1, 3 and 4 Figs). The highest amount DNA was obtained from tail-hair samples (with a maximum of 25 μg), and the lowest was from whole-blood samples (with a maximum of 4 μg). Unlike DNA extracted from buccal swabs and tail-hair follicles, DNA extracted from whole-blood samples was similar both in appearance on the gel and average quantity (μg) for all five-starting whole-blood volumes used (Table 1; Fig 2). This may be due to the overall low blood volumes (20–100 μl) used in this study. In other words, differences in the amount of extracted DNA might be more apparent if larger starting volumes (100–1000μl) were used instead. The overwhelming majority of extracted samples in this study were within the accepted range of DNA purity (260/280nm ratio = 1.8–2.2) (Fig 2) for DNA sequencing and genotyping [41]. Nonetheless, four out of twenty-seven DNA samples extracted from single buccal swabs exhibited noticeable low 260/280 nm ratio. This may be related to insufficient buccal cells captured by the swab or possible contaminant from animal feed.

Overall, we found that a single buccal swab offered a DNA amount equivalent to 100 μl of whole-blood, however, DNA extracted from only 20–30 hair follicles was greater than that extracted from five buccal swabs combined. Given the relative ease of obtaining this number of tail-hair follicles, this argues for it being superior to both whole-blood and saliva as camel DNA source.

The nonparametric ANCOVAs detected no significant interaction effect between starting DNA sample quantity and the replica factor for each DNA source (whole-blood, saliva, and tail-hair follicles) (S1–S3 Tables). This indicates that the relationship between DNA sample quantity (of each DNA source) and the extracted DNA amount (of the first elution) is similar across experimental replicates, which suggests consistency in the extraction protocol in all examined DNA sources.

In addition, the nonparametric ANCOVAs detected no significant interaction effect between starting DNA sample quantity and the individual camel factor for whole blood (S1 Table), but a significant interaction effect between starting DNA sample quantity and the individual camel factor was found for each of saliva (S2 Table) and tail-hair (S3 Table). This indicates that among all examined DNA sources, only whole-blood shows no significant difference in the relationship between starting DNA sample quantity and extracted DNA amount (of the first elution) among individual camels. The result for saliva and tail-hair was in accordance with previous studies. For example, the variability among individual camels in extracted DNA amount from different starting amounts of buccal swabs could be explained by the different number of cells captured from each swab (e.g. see DNA quantity ranges in [42–44]). Similarly, the variability among individual camels in extracted DNA amount from different starting amounts of tail-hair follicles could be explained by morphological variation in hair follicles between camel types, age, or sex [45].

Because buccal swab and the tail-hair follicles are commonly stored at room temperature (20–25°C) prior to DNA extraction, and thus are expected to experience considerable DNA degradation, we assessed and compared their rate of degradation. The rate of DNA degradation over the examined 9-month period was similar for both saliva and tail-hair (Fig 5), however, greater deterioration in the quality and purity of DNA obtained from saliva was observed, when compared to tail-hair (Fig 4). This deterioration might be due to the sensitivity of cells in saliva to degradation when stored at room temperature, or even at 4°C [46]. In contrast, DNA quantity from thirty tail-hair follicles averaged ~5 μg and ~2.0 (260/280 ratio) after being stored for 9 months. When considering all factors together, we conclude that tail-hair follicles are the best DNA source for biobanking camel specimens, given the extracted DNA quantity and quality, as well as the ease of collection, storage, and transportation.

### Prospects and conclusions

Blood and saliva are the most used sources of DNA for genetic studies, especially in humans [47], cats [48], and dogs [44]. However, our results indicate that tail-hair follicles seem to be more optimal than these two sources for large-scale genetic studies in camels especially for its ease of collection, transport, and storage prior to DNA extraction and for providing sufficient DNA amounts for genotyping and sequencing. Camel hair was previously used for STR genotyping [30, 49] and DNA sequencing [19, 21]. Beyond the currently-employed PCR applications, STR genotyping, and targeted sequencing, based on our results, we expect that camel tail-hair can be successfully applied to current genotyping and sequencing technologies. This is driven by recent successful use of tail-hair for genome-wide SNP genotyping and association studies in cattle [50, 51], horses [52], and goats [53]. It is worth noting that adequate amounts of hair (i.e. 40–50 hair follicles) should be used to produce SNP genotype calls, as indicated in a comparative analysis of the genotyping success of horse blood and hair-root samples [54]. These recent studies increase the prospects of the utility of tail-hair for camel DNA biobanking and studies associated with this biobank.

## Supporting information

S1 Fig Electrophoretic analysis of five quantities of camel blood. (a-e) 1.5% agarose gels of DNA extracted from 20, 40, 60, 80, and 100μl of camel blood, respectively. C1-C3: Majaheem, C4-C6: Sofor, C7: Waddah. Each blood quantity in the seven camels (C1-7) was extracted three times (replicas). The presented DNA in the gel is that of the first elution (E1). The ladder used in the gels is a 100 bp molecular marker. Note: the blood of six camels (C2—C7) was extracted using three ‘replicas’ for each of the five starting amounts (20, 40, 60, 80, and 100μl). The blood sample of (C1) camel was extracted once with no ‘replicas’ and only for the amounts (20, 40, 60, 80 μl) due to sample overuse in trouble shooting experiments. Following the experiments, we discovered that incorrect reagents were used in the extraction protocol for two camels (C8 and C9), and thus these were omitted from the figure.
(TIFF)

S2 Fig DNA quantities from blood, saliva, and tail-hair follicles. (a-c) DNA amounts (μg) obtained from the first elution (100μl), second elution (100μl), and combined (total), respectively.
(TIFF)

S3 Fig Electrophoretic analysis of five quantities of camel buccal swabs. (a-e) 1.5% agarose gels of DNA extracted from 1, 2, 3, 4, and 5 of camel buccal swabs, respectively. C1-C3: Majaheem, C4-C6: Sofor, C7-9: Waddah. Buccal swabs for each quantity in each of the nine camels (C1-9) were extracted three times (replicas). The DNA in the gels is that of the first elution (E1). The ladder used in the gels is a lambda-HindIII molecular marker.
(TIFF)

S4 Fig Electrophoretic analysis of five quantities of camel tail-hair follicles. (a-e) 1.5% agarose gels of DNA extracted from 10, 20, 30, 40, and 50 of camel tail-hair follicles, respectively. C1-C3: Majaheem, C4-C6: Sofor, C7-9: Waddah. Tail-hair follicles for each quantity in each of the nine camels (C1-9) were extracted three times (replicas). The DNA in the gels is that of the first elution (E1). The two ladders used in the gels are 100 bp (left side) and lambda-HindIII molecular markers (right side).
(TIFF)

S1 Table Permutation-based ANCOVA summary tables, testing the difference in the relationship between extracted DNA amount of first elution (response variable) and starting whole-blood DNA sample quantity (covariate) among each of the following fixed factors: (a) replica, (b) individual camel, and (c) breed. *df* = degrees of freedom, *SS* = unique sums of squares, *MS* = mean square, *p* = permutation test *p*-values. Statistical significance is based on a maximum of 100,000 iterations. Significant *p*-values are in bold. Three camels were not included in the analysis (see below). The C1 camel was omitted for the whole-blood analysis because it was extracted once with no ‘replicas’ and only for 20, 40, 60, 80 μl (and not 100 μl) due to sample overuse in troubleshooting experiments, making its setup inappropriate for this statistical analysis. Following the experiments, we discovered that incorrect reagents were used in the extraction protocol for two camels (C8 and C9), and thus these were omitted from this statistical analysis.
(XLSX)

S2 Table Permutation-based ANCOVA summary tables, testing the difference in the relationship between extracted DNA amount of first elution (response variable) and starting saliva DNA sample quantity (covariate) among each of the following fixed factors: (a) replica, (b) individual camel, and (c) breed. *df* = degrees of freedom, *SS* = unique sums of squares, *MS* = mean square, *p* = permutation test *p*-values. Statistical significance is based on a maximum of 100,000 iterations. Significant *p*-values are in bold.
(XLSX)

S3 Table Permutation-based ANCOVA summary tables, testing the difference in the relationship between extracted DNA amount of first elution (response variable) and starting tail-hair DNA sample quantity (covariate) among each of the following fixed factors: (a) replica, (b) individual camel, and (c) breed. *df* = degrees of freedom, *SS* = unique sums of squares, *MS* = mean square, *p* = permutation test *p*-values. Statistical significance is based on a maximum of 100,000 iterations. Significant p-values are in bold.
(XLSX)

S4 Table Permutation-based ANCOVA summary tables, testing the difference in the relationship between extracted DNA amount of first elution (response variable) and time (covariate) for saliva DNA samples among each of the following fixed factors: (a) replica, (b) individual camel, and (c) breed. *df* = degrees of freedom, *SS* = unique sums of squares, *MS* = mean square, *p* = permutation test *p*-values. Statistical significance is based on a maximum of 100,000 iterations. Significant *p*-values are in bold.
(XLSX)

S5 Table Permutation-based ANCOVA summary tables, testing the difference in the relationship between extracted DNA amount of first elution (response variable) and time (covariate) for tail-hair DNA samples among each of the following fixed factors: (a) replica, (b) individual camel, and (c) breed. *df* = degrees of freedom, *SS* = unique sums of squares, *MS* = mean square, *p* = permutation test *p*-values. Statistical significance is based on a maximum of 100,000 iterations. Significant p-values are in bold.
(XLSX)

S6 Table Permutation-based ANCOVA summary table, testing the difference in the relationship between extracted DNA amount of first elution (response variable) and time (covariate) between the two DNA sources, saliva and tail-hair (fixed factor). *df* = degrees of freedom, *SS* = unique sums of squares, *MS* = mean square, *p* = permutation test *p*-values. Statistical significance is based on a maximum of 100,000 iterations. Significant *p*-values are in bold.
(XLSX)
