# Supplementary figures and images for: Quality and quantity of dromedary camel DNA sampled from whole-blood, saliva, and tail-hair
Source: PLoS One. 2019 Jan 31;14(1):e0211743. doi: 10.1371/journal.pone.0211743 (PMC6355012; doi:10.1371/journal.pone.0211743)

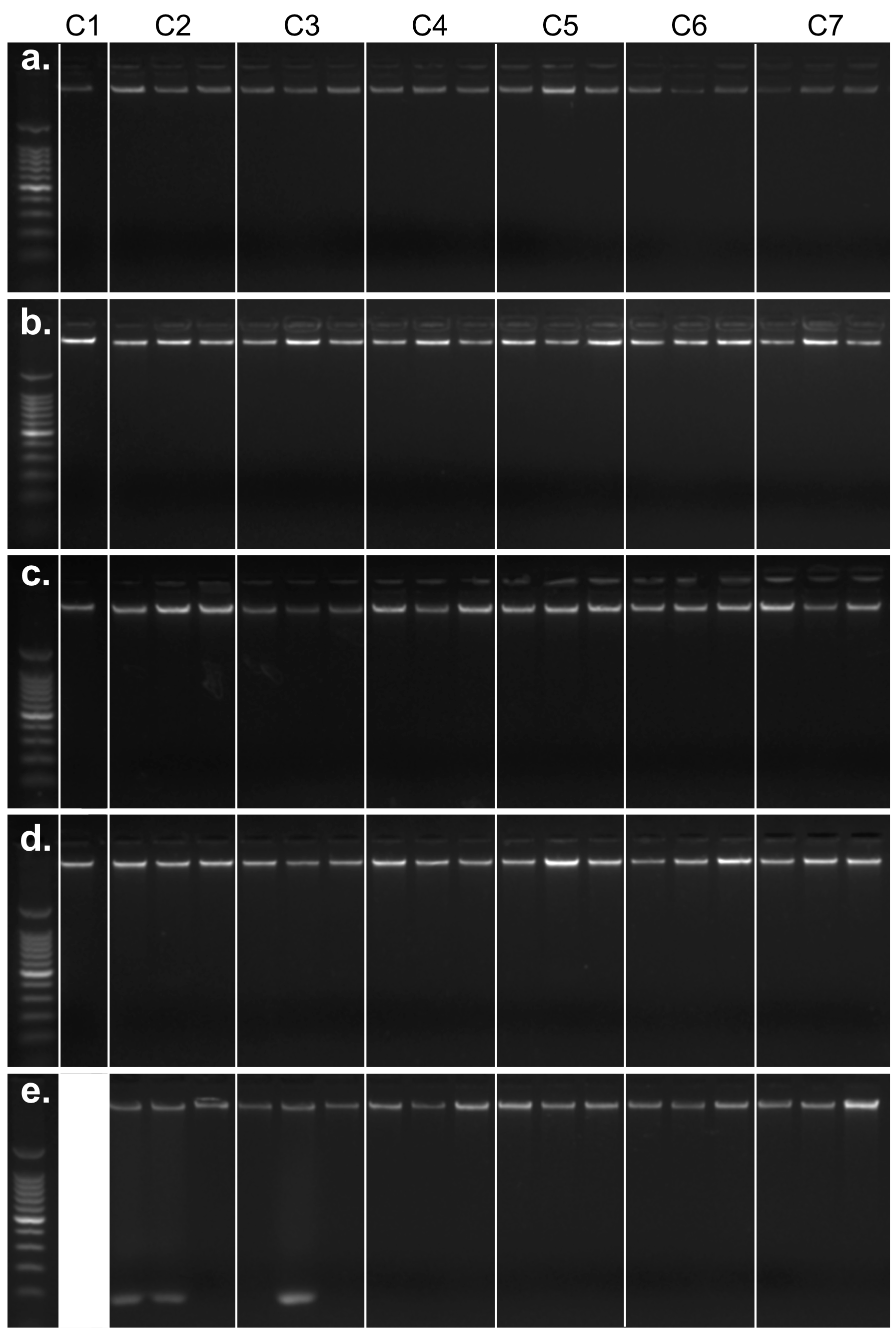

Supplement: S1 Fig — (a-e) 1.5% agarose gels of DNA extracted from 20, 40, 60, 80, and 100μl of camel blood, respectively. C1-C3: Majaheem, C4-C6: Sofor, C7: Waddah. Each blood quantity in the seven camels (C1-7) was extracted three times (replicas). The presented DNA in the gel is that of the first elution (E1). The ladder used in the gels is a 100 bp molecular marker. Note: the blood of six camels (C2—C7) was extracted using three ‘replicas’ for each of the five starting amounts (20, 40, 60, 80, and 100μl). The blood sample of (C1) camel was extracted once with no ‘replicas’ and only for the amounts (20, 40, 60, 80 μl) due to sample overuse in trouble shooting experiments. Following the experiments, we discovered that incorrect reagents were used in the extraction protocol for two camels (C8 and C9), and thus these were omitted from the figure. (TIFF) [file pone.0211743.s001.tiff]

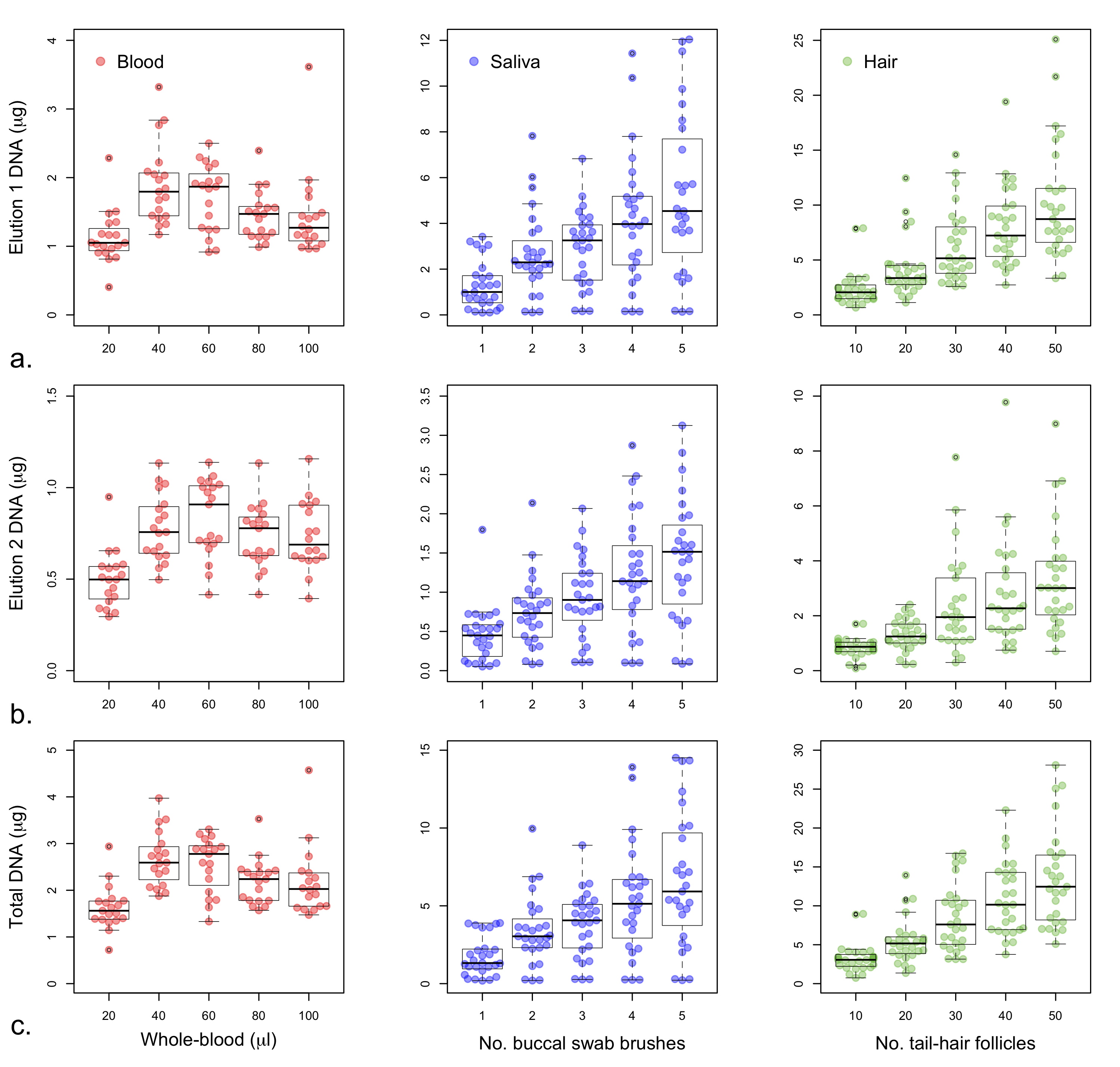

Supplement: S2 Fig — (a-c) DNA amounts (μg) obtained from the first elution (100μl), second elution (100μl), and combined (total), respectively. (TIFF) [file pone.0211743.s002.tiff]

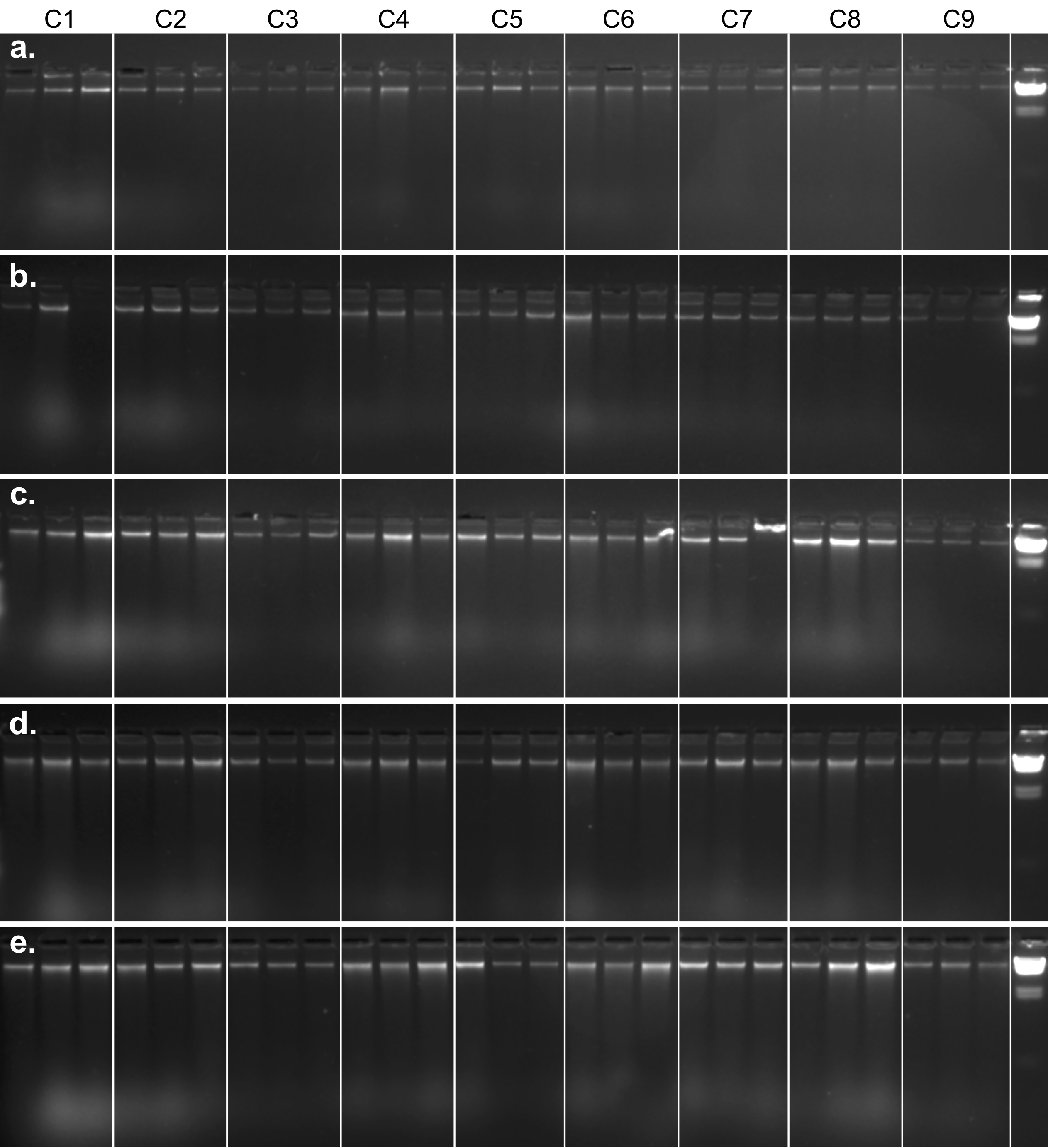

Supplement: S3 Fig — (a-e) 1.5% agarose gels of DNA extracted from 1, 2, 3, 4, and 5 of camel buccal swabs, respectively. C1-C3: Majaheem, C4-C6: Sofor, C7-9: Waddah. Buccal swabs for each quantity in each of the nine camels (C1-9) were extracted three times (replicas). The DNA in the gels is that of the first elution (E1). The ladder used in the gels is a lambda-HindIII molecular marker. (TIFF) [file pone.0211743.s003.tiff]

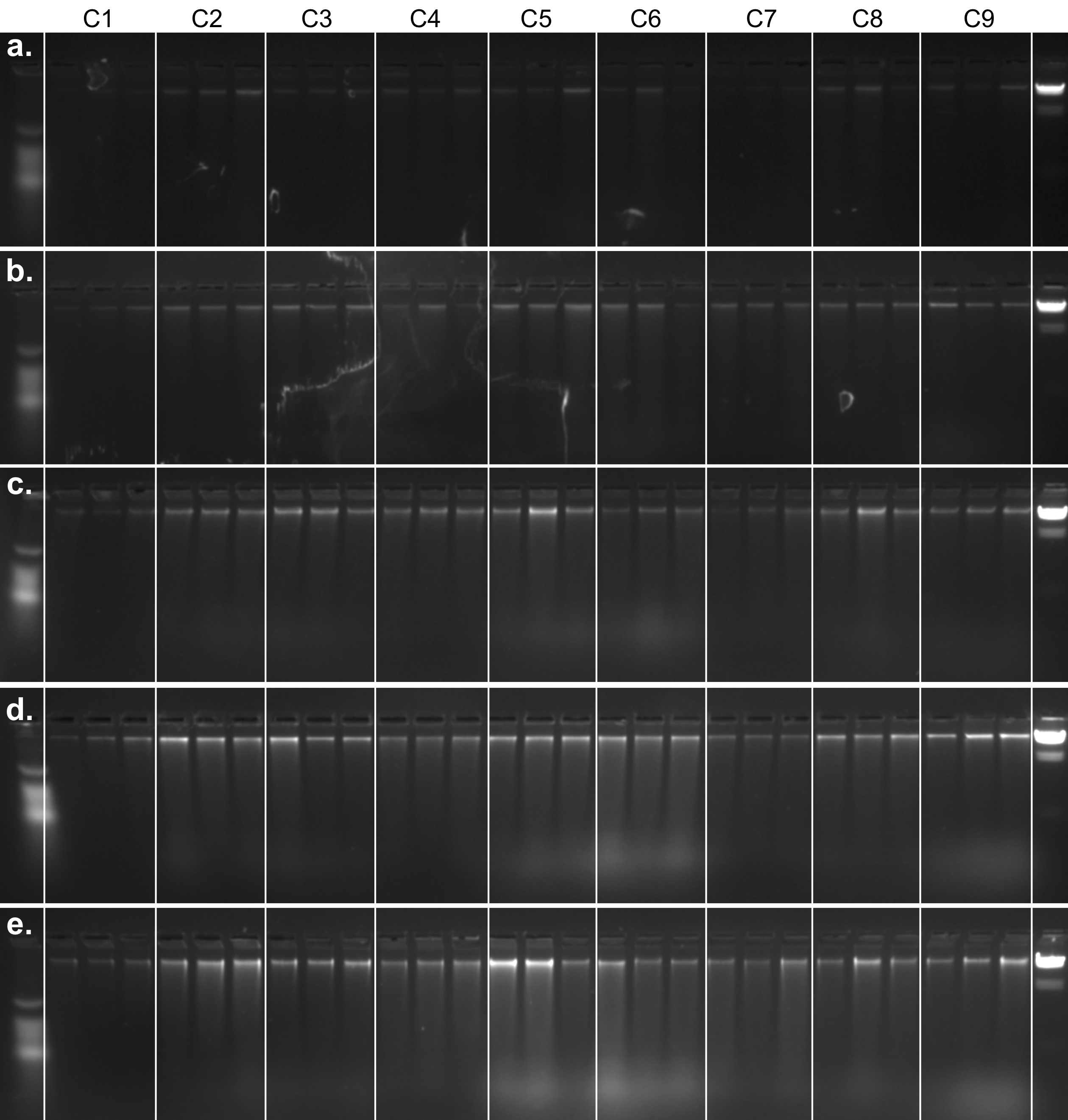

Supplement: S4 Fig — (a-e) 1.5% agarose gels of DNA extracted from 10, 20, 30, 40, and 50 of camel tail-hair follicles, respectively. C1-C3: Majaheem, C4-C6: Sofor, C7-9: Waddah. Tail-hair follicles for each quantity in each of the nine camels (C1-9) were extracted three times (replicas). The DNA in the gels is that of the first elution (E1). The two ladders used in the gels are 100 bp (left side) and lambda-HindIII molecular markers (right side). (TIFF) [file pone.0211743.s004.tiff]
